# Extracellular stimulation in neocortex lacks specificity

**DOI:** 10.17912/micropub.biology.001539

**Published:** 2025-05-28

**Authors:** Linn K. Dragsted, Shawniya Alageswaran, Aparna Suvrathan, P. Jesper Sjöström

**Affiliations:** 1 BSc Programs in Neuroscience, McGill University, Montreal, Quebec, Canada; 2 Centre for Research in Neuroscience, McGill University, Montreal, Quebec, Canada; 3 Integrated Program in Neuroscience, McGill University, Montreal, Quebec, Canada; 4 Department of Pediatrics, McGill University, Montreal, Quebec, Canada; 5 Department of Neurology and Neurosurgery, McGill University, Montreal, Quebec, Canada; 6 Department of Medicine, McGill University, Montreal, Quebec, Canada

## Abstract

By activating axons, extracellular stimulation elicits synaptic responses in patch-clamped cells. However, because axonal signaling is both orthodromic and antidromic, extracellular stimulation may lack specificity. For example, stimulating neocortical layer (L) 4 to recruit ascending inputs to L2/3 pyramidal cells (PCs) may also antidromically activate presynaptic L2/3 PCs, as their descending axons traverse L4. This contaminates L4→L2/3 activation with L2/3→L2/3 responses, which is problematic since these pathways have different properties. Using 2-photon calcium imaging, we found L2/3 PCs that responded to L4 stimulation even after synaptic blockade, demonstrating antidromic activation. Our findings highlight limitations of extracellular stimulation specificity in neocortex.

**Figure 1. L4 stimulation antidromically recruits L2/3 PCs.**
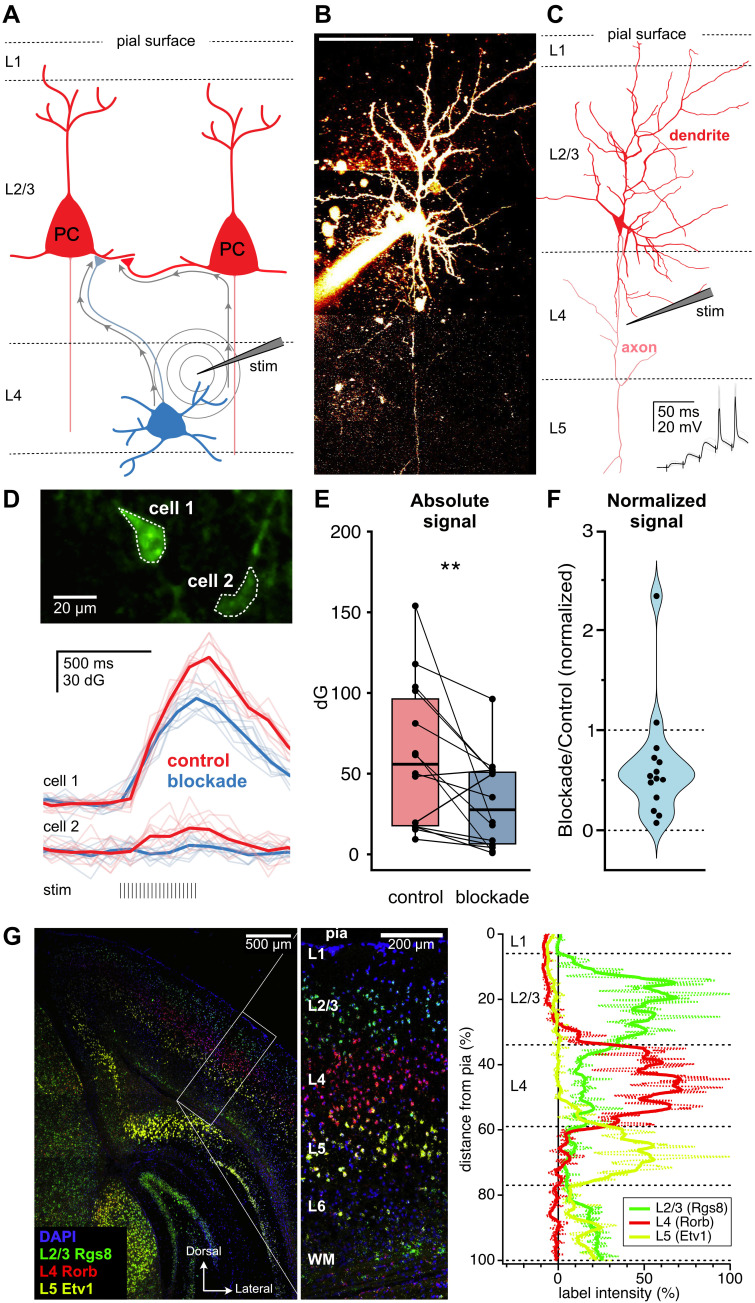
**(A)** L4 stimulation that recruits L2/3 PCs antidromically (right) should lead to poor synaptic input specificity. **(B)** Sample patched L2/3 pyramidal cell with axon descending into L4. **(C)** Morphological reconstruction shows stimulation electrode relative to descending axon as well as evoked responses, inset. **(D)** After synaptic blockade, statistically significant Ca ^2+^ transients elicited by extracellular stimulation (stim) persisted in sample cell 1 but not in sample cell 2. **(E, F)** Synaptic blockade reduced but did not abolish L2/3 PC activity, demonstrating antidromic activation of L2/3 PCs. Stimulation of L4 thus drives spiking of candidate presynaptic cells in both L4 and L2/3. **(G)** We verified upper V1 layer boundaries with *in situ* hybridization.

## Description

The strength of connections between neurons changes in response to activity through synaptic plasticity. Synaptic plasticity is thought to underlie learning and information processing in the brain. Because the rules of plasticity vary with synapse type (Larsen & Sjöström, 2015; McFarlan et al., 2023), the specificity of synapse recruitment is essential in plasticity experiments. For instance, long-term plasticity at excitatory L4→L2/3 and L2/3→L2/3 synapses relies on different molecular mechanisms (Banerjee et al., 2014), so these synapse types must be experimentally isolated when measuring plasticity.

Classically, synapses can be recruited experimentally by extracellular stimulation, which preferentially activates axonal fibers over somata or dendrites (Glasgow et al., 2019; Nowak & Bullier, 1998). For example, to study L4→L2/3 synapses in acute brain slices, a stimulation electrode is placed in L4 while responses are recorded in L2/3 (Corlew et al., 2007; Varela et al., 1997). However, L2/3 PC axons traverse L4 ( Fig. 1A- C) (Mao & Staiger, 2024), meaning some L2/3 PC activity evoked by L4 stimulation could be caused by antidromic axonal activation (Varela et al., 1997). As a consequence, it is thus possible that some synaptic responses recorded in L2/3 following L4 stimulation are due to L2/3→L2/3 synapses, rather than the intended L4→L2/3 synapses ( Fig. 1A ).

Here, we used 2-photon calcium (Ca ^2+^ ) imaging of L2/3 to look for antidromic activation of L2/3 PCs following extracellular stimulation in L4, which revealed non-specific activation of L2/3 PCs. Our findings are important for the interpretation of electrophysiological studies of neocortical synapses.

Predictably, L4 stimulation reliably elicited calcium transients in L2/3 PCs ( Fig. 1D ). However, after synaptic blockade, 12/15, or 80%, of L2/3 PCs still had statistically detectable Ca ^2+^ transients (n = 4 acute brain slices, N = 3 mice). Unsurprisingly, Ca ^2+^ transients that persisted in blockade were diminished in amplitude ( Fig. 1E, F), as expected from the absence of synaptically mediated L2/3 PC depolarization and concomitant Ca ^2+^ influx. However, the key observation here is that most Ca ^2+^ transients persisted in the face of synaptic blockade.

We were concerned that incorrect stimulation electrode placement caused unspecific L2/3 PC spiking. However, *in situ* hybridization verified layer boundaries ( Fig. 1G ), ruling out this possibility.

The frequent presence of detectable Ca ^2+^ transients in L2/3 PCs due to L4 stimulation after synaptic blockade suggests that an appreciable fraction of L2/3 PCs were antidromically recruited. This lack of specificity in recruitment of presynaptic cells by extracellular stimulation ( Fig. 1A ) suggests that more precise techniques — such as paired-patch recordings (Lalanne et al., 2016) or two-photon optogenetics (Chou et al., 2024) — are necessary to explore neocortical circuits with synapse-type specificity. Since plasticity varies across ascending L4→L2/3 and horizontal L2/3→L2/3 pathways (Banerjee et al., 2014), this lack of certainty is a key problem with extracellular stimulation, unless carefully controlled for.

One limitation of our study is that the relative contribution of antidromic L2/3 PC recruitment likely depends on many factors, such as stimulation electrode type and shape, as well as stimulation strength, frequency, and placement. It is thus not possible to assess the precise frequency of this problem in the prior literature.

The problem we highlight is particularly relevant for brain regions where axons of different cell types are intermingled, such as in neocortical circuits. For circuits where fibres are spatially segregated — such as for Schaeffer collaterals onto CA1 PCs in the hippocampus (Basu & Siegelbaum, 2015) or parallel fibres onto cerebellar Purkinje cells (D'Angelo, 2018) — this problem is likely less relevant.

In summary, we highlight here an important lack of specificity in extracellular stimulation experiments. Our study thus encourages caution in their interpretation.

**Methods**

Male Emx1 ^Cre/Cre^ mice (Jackson lab #005628) were crossed with female gCaMP6f ^flox/flox^ mice (Jackson Lab #028865). Experiments were done with postnatal day (P) 15-33 male and female offspring. Artificial cerebrospinal fluid (ACSF) containing (in mM) 125 NaCl, 2.5 KCl, 1.25 NaH _2_ PO _4_ , 26 NaHCO _3_ , 1 MgCl _2_ , 2 CaCl _2_ , and 25 glucose was prepared and adjusted with glucose to 338 mOsm. Mice were anesthetized with isoflurane and decapitated. The brain was removed and placed in chilled ACSF. 300-µm coronal slices of the primary visual cortex were obtained using a Campden Instruments 5000 mz-2 vibratome (Lafayette Instrument Company, Lafayette, IN, USA). Slices were incubated in ACSF at 33°C for 10 minutes and then kept at room temperature for >1 hour before recording.

Glass patch pipettes were pulled with a P-1000 Puller (Sutter Instrument, Novato, CA, USA). Patch pipettes (4-7 MΩ) were filled with internal solution containing (in mM) 5 KCl, 115 K-Gluconate, 10 HEPES, 4 Mg-ATP, 0.3 Na-GTP, 10 Na-Phosphocreatine, 0.05 Alexa Fluor 594, adjusted with KOH to pH 7.4 and with sucrose to 310 mOsm. Extracellular stimulation pipettes were filled with ACSF. Experiments were performed at 32-34°C using a custom-modified SliceScope (Scientifica, UK) (Abrahamsson et al., 2017).

The stimulation electrode was placed in L4. Extracellular stimulation was delivered as ten 50-V 0.1-ms-long biphasic pulses at 20 Hz using a BSI-950 stimulus isolator (Dagan Corporation, Minneapolis, MN). Two-photon excitation of gCaMP6f was achieved using a Chameleon ULTRA II Ti-Sa laser (Coherent, Santa Clara, CA) tuned to 920 nm. Using a PCIe-6374 digitization board (NI, Austin, TX, USA) and ScanImage 2021 (MBF Bioscience, Williston, VT, USA) running in MATLAB (The MathWorks, Natick, MA, USA), Ca ^2+^ signals were captured as two-second-long movies at 9.6 Hz and 256×256 pixels averaged across 10 repeats ( Fig. 1D ). To cover a column from the pia down to L4, movies were repeated at juxtaposed locations.

Synaptic blockade was achieved by washing in 200 µM D/L-AP5 and 20 µM CNQX (Hello Bio). After 5 minutes, the same locations were re-imaged.

Regions of interest corresponding to PCs with Ca ^2+^ signals were analyzed using LineScanAnalysis (10.5281/zenodo.7853953) running in Igor Pro 9 (WaveMetrics Inc., Lake Oswego, OR, USA). The Ca ^2+^ transients of each region of interest were measured as raw dG and normalized to the area immediately surrounding the region of interest.

In slice experiments, L2/3 was visually identified by the presence of relatively small PCs. L4 was defined as characteristically dark and granular. L5 was identified by the presence of conspicuous PCs with large somata and thick apical dendrites. To confirm the layer boundaries, we used *in situ* hybridization (Lein et al., 2007) to label the layers (Wang et al., 2012) in 20-µm-thick fresh frozen coronal V1 sections from P24 mice that were not used for slice experiments (RNAscope Fluorescent Multiplex V2 assay #323270, ACD Bio; L2/3: Rgs8 TSA Vivid 520 #515041; L4: Rorb TSA Vivid 650 #444271; L5: Etv1 TSA Vivid 570 #557891). Probes were imaged sequentially with a Zeiss LSM780 confocal microscope with objective LD Plan-Neofluar 20×/0.40. Layer boundaries were defined as the intersection of peak-normalized fluorescent intensity profiles of neighbouring layers ( Fig. 1G ).

L2/3 PCs were recorded in current clamp and sampled at 40 kHz using a PCIe-6323 board (NI) controlled by MultiPatch (10.5281/zenodo.7854025) running in Igor Pro 9 ( Fig. 1G ). Two-photon image stacks of patched PCs were acquired at 820 nm ( Figure 1B ).

Control and blockade values were statistically compared using Student’s *t* -test for the difference of the means ( Fig. 1E ). To ensure fair comparison, laser power and detector gains were kept the same before and after synaptic blockade. Ca ^2+^ transients were deemed detectable after synaptic blockade if a one-sample Student’s *t* -test compared to zero across the 10 repeats achieved p < 0.05 significance. Since statistical comparisons did not depend on dG normalization to baseline fluorescence, we report data as raw values ( Fig. 1D- F).

**Declarations**

**Ethics approval**

Procedures were carried out in accordance with the *Canadian Council on Animal Care* and overseen by the Montreal General Hospital *Facility Animal Care Committee* , with appropriate licenses.

**Availability of data and materials**

The datasets used and/or analysed during the current study are available from the corresponding author.

**Competing interests**

The authors declare no competing interests.
